# Gougerôt Sjögren compliqué d'aspergillome pulmonaire

**DOI:** 10.11604/pamj.2013.15.71.2405

**Published:** 2013-06-25

**Authors:** Faida Ajili, Najeh Boussetta

**Affiliations:** 1 Service de médecine interne, Hôpital militaire de Tunis, Tunisie

**Keywords:** Aspergillome pulmonaire, Gougerôt Sjögren, néphrite interstitielle, articulation, pulmonary aspergilloma, Gougerôt Sjögren, interstitial nephritis, articulation

## Image en médecine

L'incidence de l'aspergillose a récemment augmenté parallèlement au développement des traitements immunosuppresseurs et du sida. La localisation pulmonaire reste la plus fréquente. Sa survenue sur un poumon antérieurement sain, comme le cas de notre patiente, est rare. Il s'agit d'une patiente âgée de 37 ans, suivie pour un syndrome de Gougerôt Sjögren compliqué d'une néphrite interstitielle avec une insuffisance rénale chronique. Elle avait été hospitalisée pour un syndrome infectieux évoluant depuis 2 mois, un purpura vasculaire des membres inférieurs et une polyarthralgie des grosses articulations. A la radiographie du thorax, on notait une opacité hétérogène du sommet pulmonaire gauche. Le scanner thoracique avait conclu au diagnostic d'aspergillome pulmonaire avec une image en grelot. L'antigénémie aspergillaire était positive à 48 heures d'intervalle. Devant l'insuffisance rénale, l'amphotéricine B avait été contre indiquée et la patiente avait été mise sous Caspofungine (Cancidas^®^) à la dose de 70 mg à j1, puis 50 mg/j et a eu une lobectomie gauche, vue l'extension de l'image radiologique sur les radiographies de contrôle avec des suites opératoires simples. L'aspergillose pulmonaire reste une maladie grave survenant sur un terrain particulier. Son pronostic est actuellement détourné par la survenue des nouveaux anti-fongiques.

**Figure 1:**
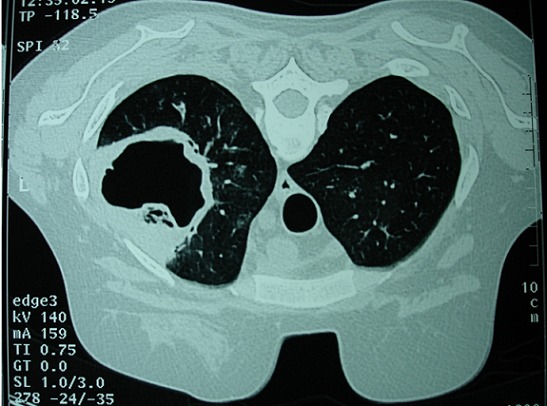
Coupe tomodensitométrique montrant une image en grelot au niveau du poumon gauche en rapport avec un aspergillome pulmonaire

